# The Inhibition of Prolyl Endopeptidase (PREP) by KYP-2047 Treatment to Reduce Myocardial Ischemia/Reperfusion Injury

**DOI:** 10.3390/antiox14040442

**Published:** 2025-04-08

**Authors:** Laura Cucinotta, Nicoletta Palermo, Alessio Ardizzone, Anna Paola Capra, Michela Campolo, Emanuela Esposito, Giovanna Casili, Marika Lanza

**Affiliations:** 1 Department of Chemical, Biological, Pharmaceutical and Environmental Sciences, University of Messina, Viale Ferdinando Stagno D’Alcontres, 31, 98166 Messina, Italy; 2 Department of Biomedical and Dental Sciences and Morphofunctional Imaging, University of Messina, Via Consolare Valeria, 1, 98125 Messina, Italy

**Keywords:** myocardial ischemia/reperfusion injury (MI/R), prolyl endopeptidase, KYP-2047, MAPK, NF-κB

## Abstract

Myocardial ischemia–reperfusion injury (MI/R) is a negative and adverse cardiovascular outcome following myocardial ischemia, cardiac surgery, or circulatory arrest. Prolyl endopeptidase (PREP) appears to be involved in inflammatory responses, so it could be a possible therapeutic target for counteracting ischemia injury. This study aimed to investigate the role of PREP inhibitor, KYP-2047 (4-phenylbutanoyl-l-prolyl-2(S)-cyanopyrolidine), in the modulation of molecular and biochemical processes involved in MI/R. MI/R was induced through coronary artery occlusion (15 min), followed by reperfusion (2 h). KYP-2047 was intraperitoneally administrated at doses of 2.5 mg/kg and 5 mg/kg 24 h before the surgical procedures. The hearts were removed and processed for analysis. KYP-2047 treatment limited ischemic myocardial-induced histological damage and neutrophil accumulation, limiting inflammation, fibrosis, and apoptosis processes. Additionally, KYP-2047 was able to modulate p-38 and p-ERK expression, suggesting an improving role in recovering cardiac function. These findings highlighted the protective effects of KYP-2047 pretreatment in MI/R injury, suggesting PREP as a potential target therapy for the pathogenesis of MI/R. Although the molecular mechanisms underlying the action of KYP-2047 are still to be explored, these results suggested that the regulation of NF-κB, apoptosis, and MAPK pathways by KYP-2047 treatment could preventatively limit the damage caused by MI/R.

## 1. Introduction

Cardiovascular diseases cause approximately one-third of fatalities worldwide. In the 21st century, ischemic heart disease represents the most common cardiovascular disease and is recognized as a significant disease for sustainable development [1]. Myocardial ischemia–reperfusion injury (MI/R) can lead to adverse cardiovascular events after myocardial ischemia, circulatory arrest or cardiac surgery and remains a challenging entity in cardiovascular medicine [2]. Vascular, metabolic, and neurohormonal factors interact and take part in causing a deficit to perfusion without the typical chest pain [3]. Comprehending these processes is essential for identifying various clinical manifestations and creating focused therapies [4]. Ischemia–reperfusion (I/R) refers to a condition where tissue is initially deprived of oxygen and nutrients (ischemia) due to a reduction or blockage in blood flow, followed by the restoration of blood flow (reperfusion). I/R damage is caused by complex molecular and cellular processes that are the result of the convergence of different biological pathways [5]. Interestingly, following MI/R, macrophages penetrate damaged cardiac tissue and modify their polarization phenotype to react to acute inflammation and chronic fibrotic remodeling [6]. Moreover, recent evidence showed that microvascular abnormalities, including compromised angiogenesis, are part of the pathogenesis of MI/R; therefore, vasopromotion to enhance collateral circulation is an important therapeutic avenue in the treatment of MI/R injury [7]. To date, there is no effective treatment for reperfusion injury, and research is increasingly focusing on finding an efficient preventive approach. Although there are many successful animal studies on how to prevent reperfusion injury, clinical translation has not always been satisfactory [8,9]. There is no pharmacological treatment even if animal models have shown positive results; this may be explained by variations between preclinical animal MI/R models and the clinical scenario in patients, including comorbidities, age, and cotreatments [10]. A lack of particular biomarkers for properly diagnosing MI/R and a limited understanding of the underlying pathophysiology might be other reasons. New molecules strongly involved in angiogenesis and inflammation have been recently evaluated in various inflammatory models. The release of proinflammatory and pro-angiogenic chemicals is facilitated by the proteolytic enzyme prolyl endopeptidase (PREP), which is a member of the serine protease family [11]. PREP is expressed in different body tissues and the cells of the renin–angiotensin system, and PREP has a significant role in peptide regulation, participating in processing angiotensin II. However, there is evidence that PREP plays a role in the digestion of thymosin β4 to generate the tetrapeptide Ac-SDKP, which increases angiogenesis and decreases fibrosis and apoptosis [12]. Considering the involvement of PREP in inflammatory and pro-angiogenic processes, PREP inhibitors may represent new therapeutic approaches. Interestingly, KYP-2047 (4-phenylbutanoyl-l-prolyl-2(S)-cyanopyrolidine) represents the most selective and potent inhibitor, having a good ability to reach PREP intracellularly [13]. KYP-2047 causes a conformational stabilization of PREP’s active site by regulating protein–protein interactions that modulate inflammation and oxidative stress [14]. It has been demonstrated that PREP inhibition has substantial potential in being able to reduce the level of inflammation, and moreover, our previous studies have demonstrated the ability of KYP-2047 to reduce MI/R damage in organs such as the intestine and kidneys, which makes PREP inhibition an interesting therapeutic approach for MI/R [15,16,17,18]. Based on this evidence, in the present study, we investigated the preventive effects of KYP-2047 in an in vivo model of MI/R.

## 2. Materials and Methods

### 2.1. Materials

KYP-2047 (Sigma, CAS No.: SML020) was purchased from Sigma-Aldrich (Milan, Italy). All other chemicals used in this study were of the highest commercial grade available. All the stock solutions were prepared in non-pyrogenic saline (0.9% NaCl, B. Braun Melsungen AG, Berlin, Germany).

### 2.2. Animals

Male adult CD1 mice (25–30 g; Envigo, Milan, Italy) were accommodated in stainless steel cages and kept on a 12 h light/12 h dark cycle at an ambient temperature of 22 °C ± 1 °C. The animals were 6–8 weeks old and had free access to standard rodent chow and water ad libitum. All efforts were made to reduce animal pain. Animal care was in conformity with current legislation for the protection of animals used for scientific purposes (D.Lgs 2014/26 and EU Directive 2010/63).

### 2.3. Myocardial I/R Surgery

All mice were anesthetized with a mixture of ketamine and xylazine, under sterile conditions. Myocardial ischemia–reperfusion injury was induced by occlusion of the left anterior descending artery (LAD) as described before by Di Paola et al. [19]. Briefly, after anesthesia, the body temperature was maintained at 37 ± 1 °C with the support of a heating pad during the surgery. In brief, the pericardium was removed after thoracotomy at the fifth intercostal space, and a 6-0 silk thread was placed around the LAD approximately 1–2 mm below its origin. After 15 min, to allow the reperfusion of the previously ischemic myocardium, the ligation was released. The duration of reperfusion was predetermined at 2 h. The identical procedure was performed on sham-operated animals, except the left anterior descending coronary artery was not tied. After surgery, mice were allowed to equilibrate under a heating lamp under observation for at least 6 h. The choice of the occlusion time and consequently the type of experimental model were chosen in the report of the literature to maximize the reproducibility of functional myocardial damage while minimizing mortality in animals. KYP-2047 was administrated to animals at doses of 2.5 and 5 mg/kg 24 h before the surgical procedures. The dose and route of administration of KYP-2047 were chosen based on previous studies [11,15,18]. Hearts were excised for histological analysis to determine the size of ischemic injury.

### 2.4. Experimental Groups

To evaluate the effect of KYP-2047 in vivo, the animals were divided into the following groups at random:

- Sham: Mice were subjected to surgery, except for coronary artery occlusion shock, and were kept under anesthesia for the duration of the experiment (N = 8).
- Sham + KYP-2047 2.5 mg/kg group: Mice were subjected to surgery, except for coronary artery occlusion shock, and were kept under anesthesia for the duration of the experiment. KYP-2047 (2.5 mg/kg 0.001% DMSO i.p) was administered 24 h before the surgical procedures (N = 8).
- Sham + KYP-2047 5 mg/kg group: Mice were subjected to surgical procedures, except for coronary artery occlusion shock, and were kept under anesthesia for the duration of the experiment. KYP-2047 (5 mg/kg 0.001% DMSO i.p) was administered 24 h before the surgical procedures (N = 8).
- Ischemia/Reperfusion: Mice were subjected to coronary artery occlusion (15 min), followed by reperfusion (2 h) plus administration of saline (N = 8).
- Ischemia/Reperfusion + KYP-2047 2.5 mg/kg group: Mice were subjected to coronary artery occlusion (15 min), followed by reperfusion (2 h) plus administration of saline. KYP-2047 was administered intraperitoneally (2.5 mg/kg 0.001% DMSO i.p) 24 h before surgical procedures (N = 8).
- Ischemia/Reperfusion + KYP-2047 5 mg/kg group: Mice were subjected to coronary artery occlusion (15 min), followed by reperfusion (2 h) plus administration of saline. KYP-2047 was administered intraperitoneally (5 mg/kg 0.001% DMSO i.p) 24 h before surgical procedures (N = 8).

The doses of KYP-2047 (2.5 and 5 mg/kg) were based on previous in vivo studies performed in our laboratories. The Sham groups treated with KYP-2047 at both doses showed neither toxicity nor improvement compared to the Sham group; therefore, their results are not shown.

### 2.5. Histological Analysis

Hearts were collected 6 h after reperfusion. The whole heart was removed and was fixed in 10% (*w*/*v*) PBS-buffered formaldehyde solution (Bio-Optica Milano S.p.A., Milan, Italy) for 24 h at room temperature, dehydrated by graded ethanol, and embedded in paraffin (Bio-Optica Milano S.p.A., Milan, Italy) as described before [20]. Then, 7 mm slices were cut from paraffin-embedded tissue. After deparaffinization, sections were stained with hematoxylin/eosin (H&E) staining to evaluate histological damage. Data were analyzed in a double-double-blind manner, that is, analyses by the pathologist who sampled hearts, performed the sectioning, and examined the pathological images. The score scale-point was chosen based on a previous study [19], using the following criteria: 0, no damage; 1 (minor), focal swelling and necrosis of the myocytes; 2 (severe), necrosis with evidence of neutrophil infiltration in the myocytes; 3 (major), necrosis with massive neutrophil infiltration. Every piece was viewed at a magnification of ×10 and ×20 (100 μm and 50 μm scale bar), and morphological changes were evaluated by two blinded investigators. All sections were evaluated using a Nikon Eclipse Ci-L microscope (Nikon Europe B.V; Stroombaan, The Netherland).

### 2.6. Masson’s Trichrome

To determine the degree of fibrosis collagen accumulation, cardiac tissue sections were stained with Masson trichrome according to the manufacturer’s protocol (Bio-Optica, Milan, Italy) as previously described [17,21]. Images were shown at 2.5×, 10×, and 20× magnification (200 µm, 100 µm, and 50 µm scale bar, respectively), using a Nikon Eclipse Ci-L microscope.

### 2.7. Toluidine Blue Staining

The number of mast cells and their degranulation in heart tissue were evaluated using toluidine blue (Bio-Optica, Milan, Italy) as previously described [22]. Briefly, after deparaffinizing, sections were soaked in water for 5 min, moved to toluidine blue for 3 min, and then blotted carefully. Sections were placed in absolute alcohol, cleared in xylene, and finally fixed on glass slides using Eukitt (Bio-Optica, Milan, Italy). Images were shown at 2.5×, 10×, 20×, and 40× magnification (200 µm, 100 µm, 50 µm, and 20 µm scale bar, respectively). The number of metachromatic stained mast cells was determined by counting in five high-power fields (40×) per section by using a Nikon Eclipse Ci-L microscope.

### 2.8. Immunohistochemistry

The immunohistochemistry analysis was performed as described in previous work [23]. The heart sections (7 µm) were incubated overnight with the primary antibody at room temperature. The used antibodies were anti-mast cell tryptase (1:100, Santa Cruz Biotechnology, Dallas, TX, USA, sc-59587) anti-nitrotyrosine (1:100, Merck-Millipore, Burlington, MA, USA, 06-284), anti-VEGF (Santa Cruz Biotechnology, Dallas, TX, USA, sc-7269; 1:100), and anti-CD34 (Santa Cruz Biotechnology, Dallas, TX, USA, sc-74499; 1:100). The slices were rinsed with PBS and then incubated with a secondary antibody for 1 h at room temperature. After incubation, the staining agent (brown DAB) was used together with the Nuclear Fast Red counterstain. Then, the sections were observed using a Nikon Eclipse Ci-L microscope. The percentage area of immunoreactivity (determined by the number of positive pixels) was expressed as a percentage of the total tissue area (red staining) within five random fields at 2.5×, 10×, and 20× magnification (200 µm, 100 µm, and 50 µm scale bar, respectively), after being quantified using ImageJ software version 1.54.

### 2.9. Terminal Deoxynucleotidyl Transferase-Mediated UTP End Labeling (TUNEL) Assay

The TUNEL staining was performed using a cell death detection kit following the manufacturer’s instructions (Roche, Basel, Switzerland, Catalog: 12156792910), as previously described [24]. The sections, after being deparaffinized and hydrated, were permeabilized with 0.1 M citrate buffer and then incubated in the TUNEL reaction at 37 °C for 60 min in the dark. For TUNEL staining, 2.5×, 10×, and 20× magnification (200 µm, 100 µm, and 50 µm scale bar, respectively) were shown.

### 2.10. Western Blot

The heart tissues were suspended in two distinct buffers to extract the cytosolic and nuclear fractions, as previously described [25]. Cytosolic lysates from the samples were utilized for SDS-PAGE, and then membranes were incubated overnight with the following primary antibodies: anti-IκB-α (1:500; Santa Cruz Biotechnology sc-1643), anti-IL-18 (1:500; Santa Cruz Biotechnology sc-7954,), anti-p-p38 MAPK (Thr180/Tyr182) (1:500; Cell Signaling, Danvers, MA, USA, 9211S), anti-p-ERK (1:500; Santa Cruz Biotechnology sc-7383), Then, membranes were incubated with peroxidase-conjugated bovine anti-mouse secondary antibody (1:1000, Jackson ImmunoResearch, West Grove, PA, USA) for 1 h at room temperature. Signals were evaluated via a chemiluminescence (ECL) detection system reagent according to the manufacturer’s instructions). The expression of the protein bands was quantified by densitometry with Bio-Rad ChemiDoc TMXRS + software 6.1 (Bio-Rad, Milan, Italy) and standardized to β-actin (1:500; Santa Cruz Biotechnology sc-47778) or ERK 1/2 (1:500; Santa Cruz Biotechnology sc-514302).

### 2.11. ELISA Assay

An ELISA kit was used to detect the levels of p65 (Cusabio, Catalog: CSB-E08789m) on cytosolic and nuclear tissue lysates according to the manufacturer’s protocols. Briefly, the samples (100 µL) were added to anti-NF-κBp65-coated well plates and incubated at 37 °C for 2 h. Subsequently, 100 µL of the horseradish peroxidase (HRP) was added to each well and incubated for 1 h at 37 °C. The plates were washed with washing buffer 1X, and TMB (3,3′5,5′ tetramethyl-benzidine) substrate solution (90 µL) was added and incubated for 15 min at 37 °C. Finally, the stop solution (50 µL) was added, and the concentrations of p65 were determined spectrophotometrically at an absorbance of 450 nm and interpolated with a standard curve.

### 2.12. Statistical Analysis

Experimental data are expressed as mean ± standard deviation (SD) of N observations, in which N represents the number of animals studied. One-way ANOVA analysis of variance followed by the Bonferroni post hoc test for multiple comparisons was used to analyze the results, and only a *p*-value less than 0.05 was considered significant. Data are representative of a minimum of three independent experiments.

## 3. Results

### 3.1. Effects of KYP-2047 on Histological Assessment

**Figure 1:**
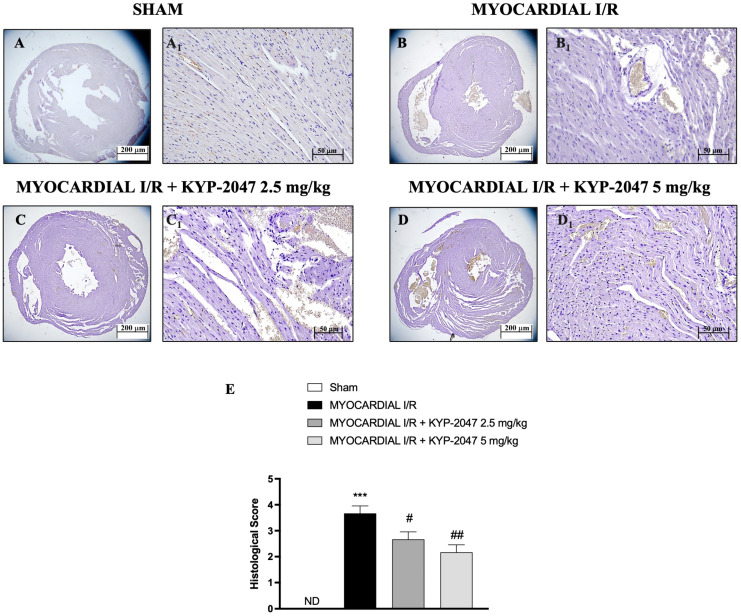
KYP-2047 reduced the severity and the degree of cell necrosis. H&E staining of Sham group (**A**,**A1**), Myocardial I/R group (**B**,**B1**), Myocardial I/R + KYP-2047 2.5 mg/kg group (**C**,**C1**), Myocardial I/R + KYP-2047 5 mg/kg group (**D**,**D1**); see histological score (**E**). Here, 2.5× and 20× magnification is shown (200 μm and 50 μm scale bar, respectively). Data are expressed as mean ± SD of N = 8 mice/group. (ND) Not detectable. *** *p* < 0.001 vs. Sham; # *p* < 0.05 vs. MI/R; ## *p* < 0.01 vs. MI/R.

H&E staining was performed to evaluate the morphology of myocardial cells. Histological examination showed tissue damage and necrosis with neutrophil infiltration in the ischemic reperfused heart (Figure 1(B,B1)) compared to the treatment (Figure 1(C,C1) and 1(D,D1); see histological score in Figure 1E). In Sham mice, myocardial tissue structure assumed the typical normal architecture (Figure 1(A,A1); see histological score in Figure 1E). The myocardial tissue in the MI/R group showed a disordered arrangement of myocardial fibers and a large number of inflammatory cell infiltrations; instead, in the MI/R + KYP-2047 group, cardiomyocytes were arranged in a more orderly manner with a decrease in necrosis; furthermore, the degree and severity of cell necrosis were significantly lower after pretreatment with KYP-2047 5 mg/kg.

### 3.2. Role of KYP-2047 Pretreatment in Reducing Fibrosis

**Figure 2:**
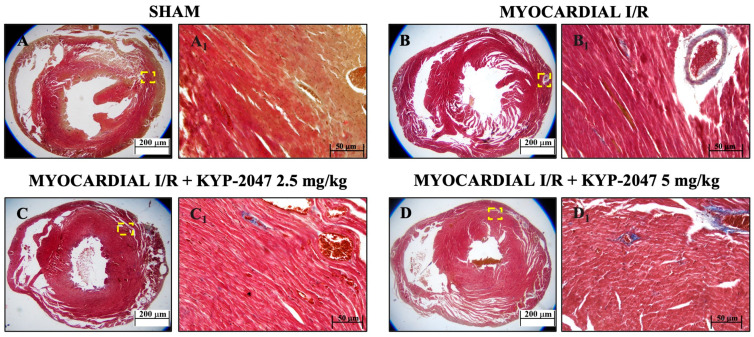
Inhibition of PREP by KYP-2047 prevented collagen deposition. Masson’s trichrome staining of Sham group (**A**,**A1**), Myocardial I/R group (**B**,**B1**), Myocardial I/R + KYP-2047 2.5 mg/kg group (**C**,**C1**), Myocardial I/R + KYP-2047 5 mg/kg group (**D**,**D1**). Red parts index cardiomyocytes and blue parts index fibrosis. Magnification 2.5×, scale bar 200 μm (**A**–**D**); magnification 20×, scale bar 50 μm (**A1**–**D1**).

Masson’s trichrome staining was used to assess mouse heart tissue. Red staining indicates healthy tissue, while blue staining indicates the area of fibrotic scarring. The data showed an increased level of collagen deposition in MI/R (Figure 2(B,B1)) because of the normal thrombus that formed in the tissue. However, KYP-2047 at doses of 2.5 and 5 mg/kg was not capable of preventing collagen deposition (Figure 2(C,C1) and Figure 2(D,D1), respectively).

### 3.3. Effect of KYP-2047 on Myocardial Injury: Mast Cell Staining and Tryptase Evaluation

**Figure 3:**
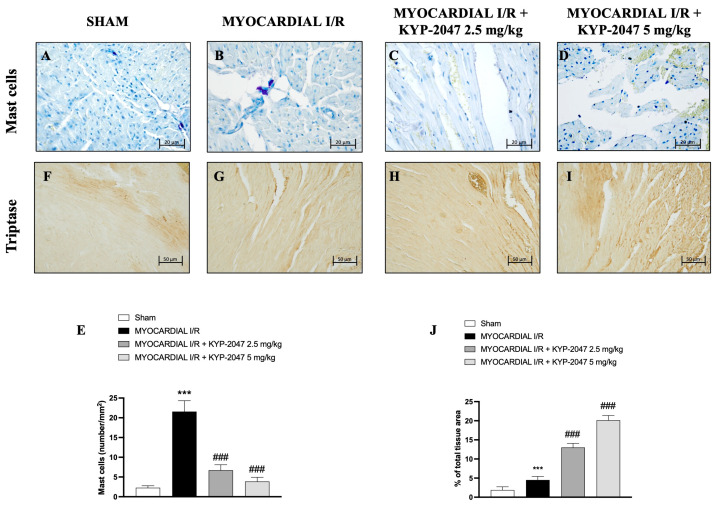
KYP-2047 reduced mast cell infiltration in heart samples. Toluidine blue staining of Sham group (**A**), Myocardial I/R group (**B**), Myocardial I/R + KYP-2047 2.5 mg/kg group (**C**), Myocardial I/R + KYP-2047 5 mg/kg group (**D**). Mast cell graph (number/mm^2^) (**E**). Immunohistochemical evaluation of tryptase. Sham group (**F**), Myocardial I/R group (**G**), Myocardial I/R + KYP-2047 2.5 mg/kg group (**H**), Myocardial I/R + KYP-2047 5 mg/kg group (**I**). See percentage of total tissue area (**J**). Magnification 40×, scale bar 20 µm (**A**–**D**); magnification 20×, scale bar 50 µm (**F**–**I**). Data are expressed as mean ± SD of N = 8 mice/group. *** *p* < 0.001 vs. Sham; ### *p* < 0.001 vs. MI/R.

The presence of mast cells in myocardial tissue was evaluated by toluidine blue staining. The data showed an increase in mast cell (MC) infiltration in the heart tissues collected from mice subjected to MI/R (Figure 3B), compared to Sham mice (Figure 3A; see percentage of total tissue area in Figure 3E). However, both doses, 2.5 mg/kg and 5 mg/kg, prevented MC infiltration in heart tissues (Figure 3C and Figure 3D, respectively) as shown by mast cell density (Figure 3E). On the other hand, the levels of tryptase (Figure 3F–I; see percentage of total tissue area in Figure 3J), evaluated by immunohistochemistry, increased following the pretreatment with the highest dose of KYP-2047 (see Figure 3I; see percentage of total tissue area in Figure 3J).

### 3.4. Effect of KYP-2047 on Angiogenesis in Myocardial I/R

**Figure 4:**
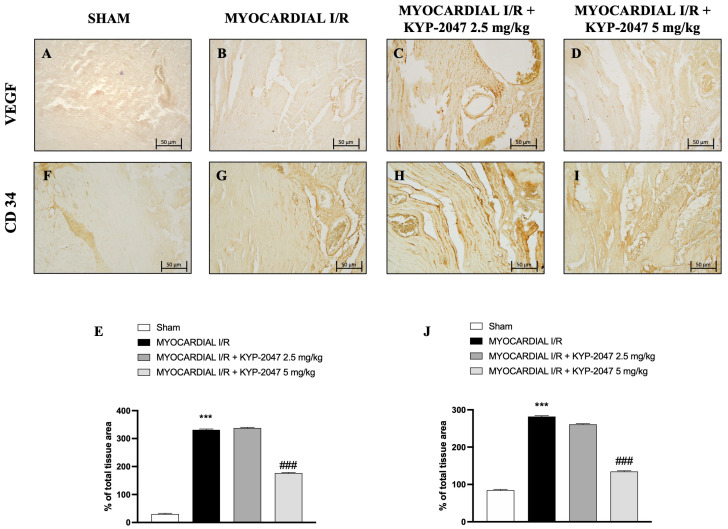
KYP-2047 treatments reduced myocardial angiogenesis. Immunohistochemistry of VEGF: Sham group (**A**), Myocardial I/R group (**B**), Myocardial I/R + KYP-2047 2.5 mg/kg group (**C**), Myocardial I/R + KYP-2047 5 mg/kg group (**D**); see percentage of total tissue area (**E**). Immunohistochemistry of CD34: Sham group (**F**), Myocardial I/R group (**G**), Myocardial I/R + KYP-2047 2.5 mg/kg group (**H**), Myocardial I/R + KYP-2047 5 mg/kg group (**I**); see percentage of total tissue area (**J**). Magnification 20×, scale bar 50 μm. Data are expressed as mean ± SD of N = 8 mice/group. *** *p* < 0.001 vs. Sham; ### *p* < 0.001 vs. MI/R.

Vascular endothelial growth factor (VEGF) expression is regulated by hypoxia and cytokines; specifically, it represents a specific endothelial cell mitogen and plays an important role in myocardial angiogenesis and vascular leakage. In heart tissue collected from mice subjected to MI/R, an increase in positive staining for VEGF was observed through immunohistochemistry analysis (Figure 4B; see percentage of total tissue area in Figure 4E), compared to control mice (Figure 4A; see percentage of total tissue area in Figure 4E). Furthermore, an increase in positive cells for CD34 in the MI/R group (Figure 4G; see percentage of total tissue area in Figure 4J), an index of the ability to stimulate angiogenesis after injury, was also observed. The data suggest that KYP-2047 at the dose of 5 mg/kg was able to reduce both VEGF- and C34-positive cells, as shown in Figure 4C,D (see percentage of total tissue area in Figure 4E) and Figure 4H,I (see percentage of total tissue area in Figure 4J), respectively.

### 3.5. Effect of KYP-2047 on Nitrosative Stress and DNA Damage

**Figure 5:**
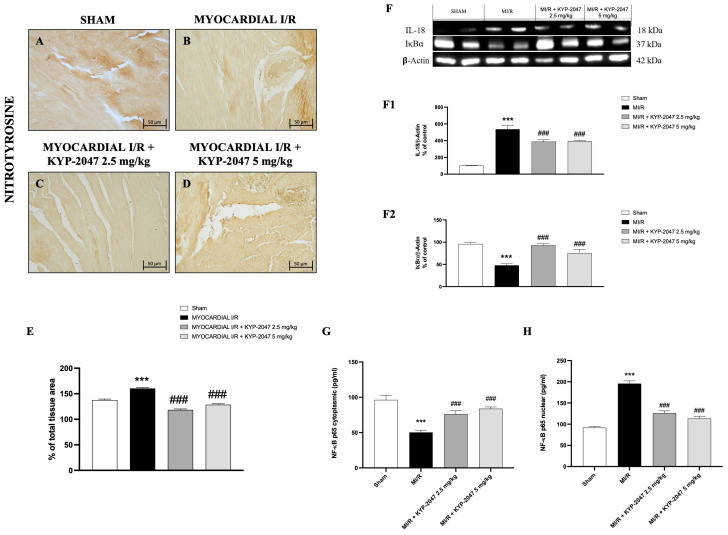
KYP-2047 treatments reduced nitrotyrosine content and modulated the NF-κBp65 pathway. Immunohistochemistry of nitrotyrosine: Sham group (**A**), Myocardial I/R group (**B**), Myocardial I/R + KYP-2047 2.5 mg/kg group (**C**), Myocardial I/R + KYP-2047 5 mg/kg group (**D**); see percentage of total tissue area (**E**). Images are shown at 20× magnification, scale bar 50 μm. Western blot analysis of IL-18 (**F**,**F1**) and IκB-α (**F**,**F2**) is shown. Actin (in (**F**)) was used as a loading control. Representative blots of three experiments are presented. ELISA assay of NF-κBp65 levels on cytosolic (**G**) and nuclear (**H**) tissue lysates is shown. Data are expressed as mean ± SD of N = 8 mice/group. *** *p* < 0.001 vs. Sham; ### *p* < 0.001 vs. Myocardial I/R (MI/R).

Nitrosative stress is closely associated with cardiovascular disease. Therefore, nitrotyrosine content, an established index of protein nitration and nitrative stress, was determined by immunohistochemistry. Compared with the control group (Figure 5A), MI/R increased nitrotyrosine content (Figure 5B), whereas the pretreatment with KYP-2047 reduced the nitrotyrosine-positive staining in heart tissue (Figure 5C,D). Furthermore, Western blot analysis showed a basal expression of IκB-α in control mice, while in mice subjected to MI/R, IκB-α expression in heart samples significantly decreased, due to the increased IκB-α cytosolic degradation (Figure 5(F,F2)). At the same time, nuclear NF-κBp65 levels were significantly increased in the MI/R group compared to Sham animals (Figure 5H). The inhibition of nuclear translocation of NF-κBp65 by IκBα phosphorylation blockade could provide an effective approach to the attenuation of MI/R injury. In fact, pretreatment with KYP-2047, at both doses, significantly reduced nuclear NF-κBp65 levels (Figure 5H). In contrast, cytoplasmic NF-κBp65 levels were reduced in the MI/R group, and treatments with KYP-2047 effectively restored these levels. Considering that IL-18 is a proinflammatory cytokine responsive to NF-κB, its expression was evaluated by Western blot analysis. The results showed increased IL-18 levels in mice that underwent MI/R compared to control mice; treatment with KYP-2047 significantly reduced IL-18 levels (Figure 5(F,F1)).

### 3.6. Effect of KYP-2047 on Apoptosis

**Figure 6:**
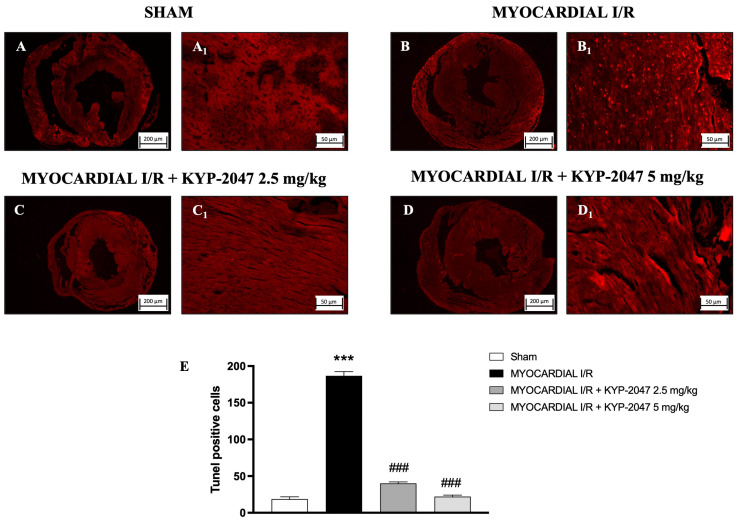
KYP-2047 reduced apoptosis process. Images of TUNEL assay are shown: Sham group (**A**,**A1**), Myocardial I/R group (**B**,**B1**), Myocardial I/R + KYP-2047 2.5 mg/kg group (**C**,**C1**), Myocardial I/R + KYP-2047 5 mg/kg group (**D**,**D1**). Count of TUNEL-positive cells (**E**). Here, 2.5× and 20× magnification is shown (200 μm and 50 μm scale bar, respectively). Data are expressed as mean ± SD of N = 8 mice/group. *** *p* < 0.001 vs. Sham; ### *p* < 0.001 vs. MI/R.

The TUNEL assay was performed to evaluate apoptosis and whether pretreatment with KYP-2047 affected cardiomyocyte I/R injury. In hearts from MI/R injury mice (Figure 6(B,B1); see positive cells in Figure 6E), the TUNEL assay showed an intense upregulation in apoptotic cells as compared to the Sham group (Figure 6(A,A1); see positive cells in Figure 6E), whereas treatment with KYP-2047 at both doses, 2.5 mg/kg and 5 mg/kg, showed a reduction in the apoptosis process (Figure 6(C,C1) and Figure 6(D,D1), respectively; see positive cells in Figure 6E).

### 3.7. Role of KYP-2047 in MAPK Pathway During MI/R

**Figure 7:**
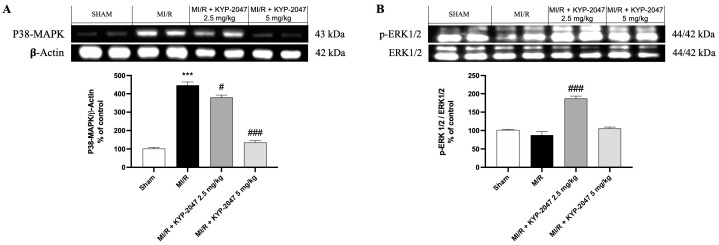
Inhibition of PREP by KYP-2047 prevented p38 activation and induced ERK1/2 phosphorylation. Western blot analysis of p38 (**A**) and Erk1/2 (**B**) phosphorylation. Whole myocardial lysate of indicated groups was processed for analysis of P-p38 and pERK1/2. Actin (in (**A**)) and total ERK1/2 (in (**B**)) were used as loading controls. Representative blots of three experiments are presented. Data are expressed as mean ± SD of N = 8 mice/group. *** *p* < 0.001 vs. Sham; # *p* < 0.05 vs. MI/R; ### *p* < 0.001 vs. MI/R.

Numerous protein kinase families are activated by myocardial ischemia–reperfusion. Among the principal protein kinase pathways linked to myocardial ischemia, there are MAP kinases, ERK 1/2, JNK 1/2, and p38 MAPKa/b. In this study, pretreatment with KYP-2047 was able to reduce p-38 expression compared to MI/R damage, suggesting an improving role in recovering cardiac function (Figure 7A). Erk 1/2 belongs to the family of serine–threonine kinases known as mitogen-activated protein kinases (MAPKs). In the context of ischemia–reperfusion, the ERK 1/2 cascade can be triggered, which can mediate cellular protection. In our study, ERK 1/2 levels were slightly reduced, but not significantly in the MI/R group compared to the control, while pretreatment with KYP-2047 at the dose of 2.5 mg/kg significantly increased ERK 1/2 phosphorylation, suggesting its cardioprotective role (Figure 7B).

## 4. Discussion

Myocardial ischemia–reperfusion injury is characterized by the death of cardiomyocytes due to reactive oxygen species (ROS) accumulation, calcium overload, and inflammation during the restoration of perfusion and simultaneous reoxygenation [26]. In its traditional form, tissue hypoxia is brought on by an embolus that obstructs the arterial blood supply, severely unbalancing the supply and demand for metabolism processes [27]. ROS overproduction, oxidative/nitrosative stress, and inflammatory reactions are some of the intricate and systematic networks at the base of the complex mechanisms of MI/R injury [28]. Moreover, apoptosis and necrosis of cardiomyocytes occur when there is cardiac ischemia, resulting in extensive myocardial tissue damage. Due to the limited proliferative capacity of myocardial cells, damaged areas of heart tissue cannot be effectively repaired or regenerated [27]. By restoring coronary artery blood flow to the ischemic tissue, timely reperfusion can effectively prevent or delay the progression of the disease and cell death.

Therefore, given the effects due to damage from MI/R, to evaluate whether inflammation, vascular alterations, and the apoptosis process involved in the pathophysiology of MI/R can be prevented, in this study, the protective effect of KYP-2047, a selective inhibitor of PREP, a serine protease involved in the release of pro-angiogenic molecules, was investigated in MI/R damage.

Inflammation contributes to the pathophysiology of cardiac IR injury; particularly, the coronary endothelium is associated with MI/R injury by activating the immune system through the expression of chemokines, cytokines, and adhesion molecules [5]. Immune cells participate in the pathogenesis of MI/R injury by triggering inflammatory responses such as monocytes, dendritic cells, and neutrophils accumulating at the damaged site [29]. Neutrophil activation and leukocyte infiltration lead to further cytokine secretion, oxidative stress, and protease release, exacerbating myocardial injury and death [30]. Pretreatment with KYP-2047 at the dose of 5 mg/kg significantly reduced the severity and the degree of cell necrosis, resulting in a better architectural state of the myocardial tissue compared to the injured one. Heart tissue was more compact and better organized with a reduced site of damage.

Furthermore, following the initial phase of ischemic insult, there is an increase in ROS as a result of oxidative stress and neutrophil clogging, and there is an obstruction of normal blood flow in capillaries and microcapillaries in a process known as the “no-reflow” phenomenon [31]. ROS generation partially contributes to mast cell degranulation, which mediates I/R, through a bilateral process [32]. Particularly, many recent studies have demonstrated the role of MCs in I/R damage such as in the heart and in the brain [33]. From bone marrow tissue, mast cells travel via the circulatory system to the target tissue, where they finish maturing and differentiating [34]. Through degranulation and the release of tryptase, chymase, and histamine, mast cells function as immediate reactors in the early stages of I/R occurrence [35]. Strong stimulation, such as myocardial ischemia, activates myocardial cells and releases cytotoxic mediators such as IL-6, TNF-α, and IL-1β [36]. The protective effect of KYP-2047, at the dose of 2.5 mg/kg and 5 mg/kg, consisted in slowing down MC degranulation, although immunohistochemistry analysis showed that KYP-2047 was not able to reduce tryptase release in the damage site. In both healthy and pathological circumstances, ROS and reactive nitrogen species (RNS) play a crucial role as intracellular and intercellular signal molecules in preserving proper homeostasis. Because of ischemic damage, in addition to hypoxia and oxidative stress, there is also nitrosative stress [37]. The formation and scavenging pathways of RNS overlap with those of ROS, which are involved in oxidative stress. These pathways are reciprocally regulated by ROS and nitric oxide (NO) [38,39]. Tyrosine nitration is the main characteristic of nitrosative stress, which causes cell membrane damage, lipid peroxidation, DNA strand breaks, the inactivation of functioning enzymes, and the activation of cascade signal responses that lead to cell death. So, high levels of ROS and RNS can damage DNA and lead cells to death. In this research, the role of KYP-2047 in preventing the increase in nitrotyrosine, a specific marker of the peroxynitrite-induced tyrosine nitration process, was confirmed in protecting cells from death. As a natural host defense mechanism, blood vessel formation in response to tissue ischemia aims to preserve tissue perfusion, which is essential for physiologic organ function. Neoangiogenesis after tissue ischemia has been associated with peripheral blood circulating progenitor cells derived from bone marrow [40,41]. In this context, CD34+ cells play paracrine functions through angiogenesis and direct incorporation into the growing vascular system. These functions include immunomodulatory, anti-inflammatory, and anti-apoptosis roles aimed at the formation of microcirculation [42,43,44]. In hypoxic conditions, other effects occur during MI/R injury: increased vascular permeability, inflammation, endothelial cell activation, an imbalance between vasodilatory and vasoconstrictor factors, and the activation of the complement and coagulation systems [27]. VEGF is an important angiogenesis factor acting exclusively on endothelial cells [45]. After damage, it plays an important role in promoting blood vessel growth and remodeling processes [46]. Thus, after myocardial damage, there were high levels of CD34+ and VEGF+ cells, as confirmed by immunohistochemistry analysis. Pretreatment with KYP-2047 at the highest dose was able to reduce VEGF levels because of the reduced damage and therefore the reduced necessity to create new vessels.

It is known that NF-κB promotes oxidative-stress-induced necrosis and I/R injury [47]. Thus, myocardial protection may be provided through inhibition of the NF-κB pathway [48,49,50,51,52]. In fact, NF-κB is maintained in an inactive form in the cytoplasm due to its binding to IκBα, preventing its translocation into the nucleus. Nevertheless, phosphorylation-induced proteolytic degradation of IκBα occurs during the activation of NF-κB signaling [53]. In this study, the results confirmed that NF-κB was activated in the heart after MI/R, while pretreatment with KYP-2047 significantly reduced the nuclear translocation of NF-κB; on the other hand, the expression of IκBα was increased even at the lowest dose of KYP-2047. Furthermore, it is known that the binding of IL18 to its receptor and the activation of NF-κB mediates the activity of IL18, leading to the production and release of numerous cytokines, chemokines, and cell adhesion molecules. Here, it was confirmed that KYP-2047 reduced both the downregulation of NF-κB and the levels of IL-18 proinflammatory cytokines.

The role of NF-κB in myocardial I/R results not only in the release of proinflammatory factors but also in promoting apoptosis of cardiomyocytes [54]. Programmed cell death has been linked to numerous heart pathologies, including myocardial ischemia through tissue damage and malfunctions, although reperfusion can prevent cell death that follows ischemia [29]. In this study, it was confirmed that pretreatment with KYP-2047 reduced the apoptosis process compared to the MI/R group.

Phosphorylation at the end of the cascade, composed of MAPK kinase (MKK) and MKK kinase (MEKK), controls p38 activity.

Myocardial ischemia leads to the activation of several protein kinase pathways, including MAPKs. In particular, phosphorylation at the end of the MAPK cascade, composed of MAPK kinase (MKK) and MKK kinase (MEKK) controls the activity of p38, an important pro-apoptotic mediator in cardiac myocytes [55,56,57,58]. The apoptosis process is also linked to the activation of ERK1/2 phosphorylates substrates in the cytoplasm or nucleus [59]. In the literature, the role of ERK1/2 is controversial; while some research has indicated that ERK1/2 expression and activation might exacerbate I/R, other research has suggested that ERK1/2 activity may protect against MI/R [60,61,62]. Although our study demonstrated that the pretreatment with KYP-2047 at the highest dose had a protective role and promoted the repair of damaged tissue, by inhibiting the activation of p38 MAPK, we did not observe significant changes in ERK1/2 expression at this dose. However, at lower doses of KYP-2047, we still observed a protective effect, suggesting that the activation of survival kinases such as ERK1/2 may exert a protective action against cardiac cells and reduce apoptosis as confirmed by the TUNEL assay.

There are no certain data on the possible PREP mechanisms of action; therefore, it would be interesting to provide an extracellular mechanism of action of PREP in the myocardial context, although ours could be only a hypothesis. In fact, since PREP is able to hydrolyze and activate short peptides, we could hypothesize that it is involved in the activation of atrial natriuretic peptides (ANPs), which are overexpressed following stress; specifically, ANPs are rapidly secreted from cardiomyocytes during ischemia brought on by coronary artery occlusion, playing an important role in regulating blood pressure and volume through its natriuretic and vasodilatory effects [63,64,65,66]. Therefore, PREP could promote the activations of ANPs, contributing to the exacerbation of ischemic damage; however, additional experiments would be needed to confirm our hypothesis.

## 5. Conclusions

In conclusion, this study highlighted the ability of KYP-2047 to reduce the harmful consequences of MI/R injury, suggesting PREP as a potential target therapy for the pathogenesis of MI/R. Although the molecular mechanisms underlying the action of KYP-2047 are still to be investigated, as MI/R is a complex interaction of different pathways, these findings indicated that the modulation of NF-κB, apoptosis, and MAPK pathways was able to increase cell survival, also improving cardiac function. Therefore, PREP’s multifaceted mechanism may provide more comprehensive protection against MI/R injury compared to many existing single-target drugs. The use of KYP-2047 could be particularly useful for patients with risk factors for myocardial ischemia or those undergoing cardiac procedures [67]. However, the clinical translation of drugs for MI/R injury remains a significant challenge, with many promising treatments failing to demonstrate efficacy in human trials. Additionally, further investigations are needed to define how long the protective therapy must be applied to fully prevent myocardial damage and to thoroughly assess its safety profile in clinical settings.

## Data Availability

The data presented in this study are available on request from the corresponding author.

## Abbreviations

The following abbreviations are used in this manuscript:

MI/R	Myocardial ischemia–reperfusion injury
PREP	Prolyl endopeptidase
LAD	Left anterior descending artery
H&E	Hematoxylin/eosin
TUNEL	Terminal deoxynucleotidyl transferase-mediated UTP end labeling
MCs	Mast cells
VEGF	Vascular endothelial growth factor
MAPKs	Mitogen-activated protein kinases
ROS	Reactive oxygen species
RNS	Reactive nitrogen species
NO	Nitric oxide
